# Morphometric analysis and redox state of the testicles in nandrolone decanoate and swimming treated adult male rats

**DOI:** 10.1186/s12610-021-00134-8

**Published:** 2021-07-15

**Authors:** Jasmina Sretenovic, Jovana Joksimovic Jovic, Ivan Srejovic, Vladimir Zivkovic, Katarina Mihajlovic, Milica Labudovic-Borovic, Svetlana Trifunovic, Verica Milosevic, Dejan Lazic, Sergey Bolevich, Vladimir Jakovljevic, Zoran Milosavljevic

**Affiliations:** 1 Faculty of Medical Sciences, Department of Physiology, University of Kragujevac, Svetozara Markovica 69, 34 000 Kragujevac, Serbia; 2 Faculty of Medical Sciences, Department of Pharmacy, University of Kragujevac, Svetozara Markovica 69, 34 000 Kragujevac, Serbia; 3 Institute of Histology and Embryology “Aleksandar Đ. Kostić” Faculty of Medicine, Supremalab Diagnostics and Research, University of Belgrade, 11000 Belgrade, Serbia; 4 Department of Cytology, Institute for Biological Research “Sinisa Stankovic”- National Institute of Republic of Serbia, University of Belgrade, Bulevar Despota Stefana 142, 11000 Belgrade, Serbia; 5 Faculty of Medical Sciences, Department of Surgery, University of Kragujevac, Svetozara Markovica 69, 34 000 Kragujevac, Serbia; 6 Department of Human Pathology, 1st Moscow State Medical University IM Sechenov, Trubetskaya str. 2, Russian Federation 119992 Moscow, Russia; 7 Faculty of Medical Sciences, Department of Histology and Embryology, University of Kragujevac, Svetozara Markovica 69, 34 000 Kragujevac, Serbia

**Keywords:** Testicles, Nandrolone, Training, AR, Redox state, Testicules, Nandrolone, Entraînement, AR, état redox

## Abstract

**Background:**

During the last decades, the abuse of anabolic androgenic steroids (AASs) has become popular among professional and recreational athletes. The abuse of AASs leads to decreased levels of sex hormones, but the available literature a gives very small pool of data regarding the effects of swimming alone or combined with AASs on testicle tissue. The aim of this study was to investigate the effects of four-week administration of nandrolone decanoate and swimming training alone or in combination on morphometric parameters, androgen receptor (AR) and redox state in testicle tissue. The study included Wistar albino male rats, 10 weeks old, classified into 4 groups: control (T-N-), nandrolone (T-N+), swimming training (T+N-) and swimming training with nandrolone (T+N+). The rats from nandrolone (N+) groups received nandrolone decanoate 20 mg/kg b.w.once per week. The rats from training (T+) groups, swam 1 h/day 5 days/week. The isolated testicles were measured, left testicles were routinely processed for histological analysis, while right testicles were homogenized and prepared for the analysis of the following oxidative stress biomarkers: index of lipid peroxidation (TBARS), nitrites, catalase, superoxide dismutase (SOD), and reduced glutathione (GSH).

**Results:**

Diameter, as well as cross-section area of seminiferous tubules were decreased by 10 % and 21 % (respectively) in the T-N+ group and by 15% and 41 % (respectively) in the T+N+ group compared to control. Interstitium of the testicles was decreased in all experimental groups. Reduction of immunoreactivity of AR in T-N+ group was 22 %, in T+N+ group was 9 % compared to control. TBARS levels were increased in T+N- and T+N+ groups. Nitrites were decreased in T+N+ group. Catalase activity was increased in all experimental groups. Swimming alone or combined with nandrolone decreased the level of GSH compared to control. SOD activity was decreased in T-N+ and T+N+ groups compared to control.

**Conclusions:**

Nandrolone alone or combined with swimming decreased morphometric parameters and amount of AR in testicle tissue. Changes in the redox state indicate reproductive dysfunction.

## Résumé

**Contexte:**

Au cours des dernières décennies, l’abus de stéroïdes androgéniques anabolisants (SAA) est devenu populaire parmi les athlètes professionnels et récréatifs. L’abus des SAA conduit à une diminution des niveaux d’hormones sexuelles, mais la littérature sur les effets de la natation seule ou combinée avec des SAA sur les tissus testiculaires est encore très limité. Le but de cette étude était d’étudier les effets de l’administration de quatre semaines de décanoate de nandrolone et de l’entraînement à la natation seuls ou en combinaison sur les paramètres morphométriques, le récepteur aux androgènes (RA) et l’état redox dans le tissu testiculaire. L’étude a inclus des rats mâles Wistar albinos, âgés de 10 semaines, classés en 4 groupes: contrôle (T-N-), nandrolone (T-N+), entraînement à la natation (T+N-) et entraînement à la natation avec nandrolone (T+N+). Les rats des groupes nandrolone (N+) ont reçu du décanoate de nandrolone 20 mg/kg p.c. une fois par semaine. Les rats des groupes entraînement (T+) nageaient 1 h/jour 5 jours/semaine. Les testicules isolés ont été mesurés, les testicules gauches ont été systématiquement traités pour l’analyse histologique tandis que les testicules droits ont été homogénéisés et préparés pour l’analyse des biomarqueurs de stress oxydatif suivants: indice de peroxydation lipidique (TBARS), nitrites, catalase, superoxyde dismutase (SOD) et glutathion réduit (GSH).

**Résultats:**

Le diamètre, ainsi que la section transversale des tubules séminifères ont été réduits de 10 % et 21 % (respectivement) dans le groupe T-N+ et de 15 % et 41 % (respectivement) dans le groupe T+N+ par rapport au groupe témoin. L’interstitium des testicules était diminué dans tous les groupes expérimentaux. La réduction de l’immunoréactivité de RA dans le groupe T-N+ était de 22 %, dans le groupe T+N+ était de 9 % par rapport au groupe témoin. Les niveaux de TBARS ont augmenté dans les groupes T+N- et T+N+. Les nitrites ont diminué dans le groupe T+N+. L’activité de la catalase a été augmentée dans tous les groupes expérimentaux. La natation seule ou combinée à la nandrolone a réduit le niveau de GSH par rapport au contrôle. L’activité de la SOD était diminuée dans les groupes T-N+ et T+N+ par rapport au contrôle.

**Conclusions:**

La nandrolone seule ou combinée à la natation a diminué les paramètres morphométriques et la quantité de RA dans le tissu testiculaire. Les changements de l’état redox indiquent un dysfonctionnement de la reproduction.

## Background

During the last decades, many professional and recreational athletes used various types of prohibited substances in order to improve their physical performance despite the fact that their usage is prohibited since 1976 [1, 2]. The most frequently misused substances are anabolic androgenic steroids (AASs) and today this abuse has almost epidemic proportions. AASs represent a large group of synthetic derivatives of the male sex hormone testosterone and nandrolone decanoate is one of them. Nandrolone decanoate is most commonly administered in the so-called „steroid cycle” (period from 4 to 6 weeks) followed by a period of “cleaning” (period of 4 weeks) [2].

The majority of studies showed that the administration of ААSs leads to the hypertension development [3], hypertrophy of the left ventricle, accumulation of extracellular collagen in both cardiac [4, 5] and skeletal muscle [6] and hepatocellular damage [7]. Administration of ААSs has deleterious effects on testicles manifested as structural changes of the seminiferous epithelium [8] and testicular atrophy [9–11]. As showed in our previous study, nandrolone led to reduced volume density of pituitary gonadotropic cells and serum levels of luteinizing hormone (LH) and follicle stimulating hormone (FSH) [12].

Androgen receptor (AR) is a nuclear receptor localized in the cytoplasm or nucleus of the target tissue [13] and it is directly related to testosterone activity. When testosterone binds to the androgen receptor in the cytoplasm, it is then translocated to the nucleus. As a result, activation of the DNA and transcription of the specific proteins occurs, which leads to modifications of the intracellular activity [13]. Nandrolone decanoate mechanism of action is similar to testosterone [14], but with a higher affinity for AR binding [14]. AR is localized in male reproductive system, skeletal muscle, brain, liver, kidney, skin and adipocytes [15, 16]. Reduction of androgen receptor activity, as well as low testosterone levels adversely affect reproductive function in males [13]. Studies have shown that strength training increases the activity of the AR [2].

Regular physical activity has numerous positive effects, primarily on the cardiovasular system which is manifested through the increased physical performance, muscle strength and endurance. On the other hand, literature data suggest that intensive training can cause harmful effects on the testicle tissue [17], but these effects depend on the type and duration of training. Depending on the type of physical activity, serum testosterone levels, as well FSH serum levels may be elevated [12] or reduced [18–20].

Oxidative stress, nowadays named as “disorder of redox signal and control” [21, 22] represents a misbalance between overproduction of reactive oxygen species (ROS) and the ability of the enzyme system of antioxidative defense to eliminate free radicals. It is well known that the abuse of AASs, but also exercise, may disturb redox homeostasis. There are numerous literature data describing the deleterious effects of AASs abuse on the redox state on many organ systems [22]. Pathophysiological processes in the heart, liver and kidneys are mainly associated with increased oxidative stress [22]. Previous studies reported that overproduction of ROS may lead to male infertility causing disturbed steroidogenic activity in testicles and also affecting the cellular membrane macromolecules [17]. Furthermore, the testicle membrane has a high content of polyunsaturated fatty acids [23, 24] and it is known that ROS causes their damage [17]. Intensive exercise, on the other hand, changes redox homeostasis in the testicle tissue [17]. However, there are not enough data in the literature about the combined effects of AASs and swimming training on testicular morphology, amount of AR in testicles and redox state in the testicles.

Having in mind all mentioned above, our study was aimed to investigate the effects of four week long administration of nandrolone decanoate alone, swimming training alone or in their joined administration on histomorphometric parameters, amount of AR and redox state in testicle tissue.

## Material and methods

### Experimental animals

This study was performed on 32 male Wistar albino rats, 10 weeks old, weighing 200-250 g. The rats were obtained from the Military Medical Academy, Belgrade, Serbia. Rats were housed in plexiglass transparent cages (four rats per cage), at room temperature 23 ± 1 °C with 12:12 h light and dark cycles. Food and water were provided ad libitum.

The rats were randomly classified into four groups, each with 8 animals:

1. T-N-, sedentary rats without administration of nandrolone decanoate and training (control group) (T denotes training; N denotes nandrolone),
2. T-N+, sedentary rats with the administration of nandrolone decanoate (nandrolone group),
3. T+N-, swimming training rats (swimming training group),
4. T+N+, swimming training rats with the administration of nandrolone decanoate (swimming training with nandrolone group).

### Experimental protocol

The rats from swimming training groups swam in a glass pool, dimension 120 × 80 × 50 cm (length/width/height) in which the depth of the water was 60 cm. The swimming was performed every day at 9 a.m. at a water temperature of 37 °C. The first week was a period of adaptation to swimming in which the rats started with 10 min of continuous swimming and swimming time was gradually increased daily for 10 min every day until they reached the 60 min mark at the end of the week [25]. After a period of adaptation, rats were swimming 1 h per day, 5 days per week during four weeks. The rats from nandrolone groups received nandrolone decanoate (DECA DURABOLIN®, Organon, Holland) in a dose of 20 mg/kg body weight subcutaneously [12], which corresponds to the doses of AASs abused by humans [26]. In order to avoid water induced stress, rats from non-training groups were placed into the separate water tank (depth 5 cm) for five minutes at the same water temperature, as well as rats from trained groups. Upon expiry the four week of the experimental period the rats were sacrificed. The rats were sacrificed 48 h after the last swimming in order to avoid the effect of acute swimming training. Body weight of rats was measured just before sacrifice. The animals were sacrificed by cervical dislocation (Schedule 1 of the Animals/Scientific Procedures, Act 1986 UK) in short-term ketamine (Ketamine 10 %, CP-PHARMA, Burgdof, Germany; 100 mg/kg) and xylazine (Xyla, Interchemie, Holland; 10 mg/kg) anesthesia. Both testicles were isolated and measured. Gonadosomatic index was calculated according to the following formula: Gonadosomatic index = (Testicle weight)/(Body weight)x100 [24, 27]. Afterwards, the left testicles were prepared for the standard protocols for histological analysis, while the right testicles were prepared for the oxidative stress analysis.

### Tissue processing, histochemistry, immunohistochemistry and image analysis

The testicle samples were fixed, dehydrated in a series of increasing concentrations of ethanol (50-100 %), cleared in xylol and embedded in Histowax® (Histolab Product AB, Göteborg, Sweden). Paraffin blocks of testicular tissue were cut on a rotational microtome (RM 2125RT Leica Microsystems, Wetzlar, Germany). Five micrometer thick sections were prepared for quantitative histomorphometric analysis.

Androgen receptor in this study was verified by immunohistochemical staining. Tissue sections were deparaffinized and dehydrated and afterwards, sections were cooked in citrate buffer at temperature 95 C°, at pH 6.0, for 20 min. Tissue sections were cooled at room temperature for 15 min, followed by blocking endogenous peroxidase for 10 min and washing in phosphate buffer saline (PBS) for 5 min. Sections were incubated with protein block (Ultravision Protein Block, Thermo Scientific, USA) for 5 min, followed by primary antibody (Santa Cruz Biotechnology, USA) for 30 min. Testicles sections were then washed in PBS (3 × 5 min) and incubated with a Primary antibody amplifier (Qvanto, Thermo Scientific, USA) for 10 min, washed in PBS (1 × 5 min) followed by the addition of HRP (HRP Polymer Quanto) for 10 min and again washed in PBS for 5 min. Binding sites were visualized with 0.05 % diaminobenzidine (DAB; Serva, Heidelberg, Germany), after which the sections were contrasted with hematoxylin, rehydrated on increasing alcohol concentrations (70-100 %), cleared in xylene, and mounted with DPX (Sigma-Aldrich, Co., USA).

### Morphometric analysis

Morphometric analysis was used to determine the diameter and cross-section area of seminiferous tubules, the height of seminiferous epithelium and area of the interstitium of the testicles. For this analysis, we quantified only the perfect perpendicular section of the seminiferous tubules. Images were captured with a digital camera attached to the Olympus BX51 microscope (Olympus Life and Material Science Europa GmbH, Hamburg, Germany). Morphometric analysis was performed on 135–180 seminiferous tubules per animal with calibrated Axiovision software (Zeiss, USA). Interstitium areas and AR receptors were analysed by Image Pro-Plus (Media Cybernetics, USA). Briefly, for AR analyzis immunolabelled areas were segemented and black and white masks were produced showing the localization of the antigen. Results were presented in percents, in which control values were assigned as 100 % and values from experimental groups represent as an increase or decrease in comparison to the control value.

### Redox status

Isolated right testicles from all animals were measured and then frozen at -80° C. Testicles tissue were homogenized using an electrical homogenizer in PBS on ice (1/10, weight/volume at pH 7.4). The samples were centrifuged at 1200 x g for 20 min. on 4 C° [28]. Supernatant was collected for the determination of tissue values of the index of lipid peroxidation (TBARS), nitrites (NO_2_^−^), catalase (CAT), superoxide dismutase (SOD) and reduced glutathione (GSH) activity using a spectrophotometer (Shimadzu UV-1800, Japan).

### Index of lipid peroxidation (TBARS)

The degree of lipid peroxidation in the tissue homogenate was estimated by measuring TBARS using 1 % TBA (thiobarbituric acid) in 0.05 NaOH. TBA extract was obtained by combining 0.4 ml sample and 0.2 ml trichloro acetic acid; afterwards, the samples were put on ice for 10 min and centrifuged for 15 min at 6000 rpm. The samples were then incubated at 100 °C for 15 min and measured at wavelength at 530 nm. As a blind control, distilled water was used [29].

### Nitrites (NO_2_^−^)

Nitrite (NO_2_^−^) was determined as an index of nitric oxide production with Griess reagent. For NO_2_^-^ determination in tissue samples, 100 µl 3 N PCA (perchloride acid), 400 µl 20 mM ethylenediaminetetraacetic acid (EDTA) and 200 µl homogenate of testicles tissue were mixed and put on ice for 15 min and centrifuged for 15 min at 6000 rpm. After pouring off the supernatant, 220 µl K_2_CO_3_ was added. Nitrites were measured at wavelength at 550 nm. As a blind probe distilled water was used [30].

### Catalase (CAT)

CAT activity was determined according to Aebi [28]. Diluted homogenate of testicle tissue (1:7 v/v) was treated with chloroform-ethanol (0.6:1 v/v). Fifty microliters of CAT buffer, 100 µl homogenate sample, and 1 ml 10 mM H_2_O_2_ were used. The detection was performed at 360 nm wavelength and the amount of CAT was expressed as U/g tissue [31].

### Superoxide dismutase (SOD)

The activity of SOD was determined by the epinephrine method according to Beutler [32, 33]. Sample of 50 µl homogenate of testicle tissue was mixed with 1 ml carbonate buffer, and then epinephrine was added. Detection was performed at wavelength at 470 nm. The amount of SOD in testicle tissue was expressed as U/g tissue [32, 33].

### Reduced glutathione (GSH)

For determination of reduced glutathione (GSH) method based on GSH oxidation via 5,5-dithiobis-6,2-nitrobenzoic acid according to Beutler [34] was used. GSH extract was obtained by combining 0.1 ml 0.1 % EDTA, 400 µl homogenate, and 750 µl precipitation solution (containing 1.67 g metaphosphoric acid, 0.2 g EDTA, 30 g NaCl, and filled with distilled water up to 100 ml. The mixture obtained by vortex machine was put on ice for 15 min then centrifuged at 4000 rpm for 10 min. For a blind probe was used distilled water. The level of GSH was measured at wavelength at 420 nm [34].

### Statistical analysis

All data distribution was established using the Shapiro Wilk test. Statistical comparison between groups was used by one way Anova test with the post-hoc LSD test analysis for multiple comparisons. P values below 0.05 were considered statistically significant. Statistical calculations were made with the SPSS computer program, version 20.0 (SPSS Inc., Chicago, IL, USA) and data are presented as mean values ± standard deviation (SD).

## Results

### Testicles weight and gonadosomatic index

Nandrolone administration alone decreased testicles weight by 26 % (*p* < 0.05) compared to control values. Swimming training alone increased testicle weight by 36 % (*p* < 0.05) compared to nandrolone administration alone (Table 1).

**Table 1:** Average values of body weight, testicles weight and gonadosomatic index (ratio between testicle weight and body weight)

Groups	Body weight (g)	Testicles weight (g)	Gonadosomatic index (%)
T-N-	444.40 ± 5.36	1.54 ± 0.12	0.35 ± 0.02
T-N+	426.80 ± 22.33	1.14 ± 0.45 a	0.26 ± 0.10 a
T+N-	434.20 ± 11.48	1.55 ± 0.05 b	0.36 ± 0.01 b
T+N+	411.00±31.50	1.42 ± 0.06	0.34 ± 0.02 c

Results presented as mean value ± SD (*n*=8). Statistical analysis was done by using one way Anova test with the post-hoc LSD test analysis. Statistical significance between groups (*p*˂0.05) is shown as a, b, c; a: denotes control (T-N-) vs nandrolone (T-N+); b: denotes nandrolone (T-N+) vs swimming training group (T+N-); c: denotes nandrolone group (T-N+) vs swimming training with nandrolone (T+N+); (T denotes training, N denotes nandrolone) (g denotes gram)

Gonadosomatic index was decreased in T-N+ group by 26 % (*p* < 0.05) compared to control. Comparison between experimental groups, swimming training alone or combined with nandrolone showed increase of gonadosomatic index by 36 % (*p* < 0.05) and 30 % (*p* < 0.05) (respectively) in comparison to T-N+ group (Table 1).

### Morphometric analysis of the testicles

**Fig. 1:**
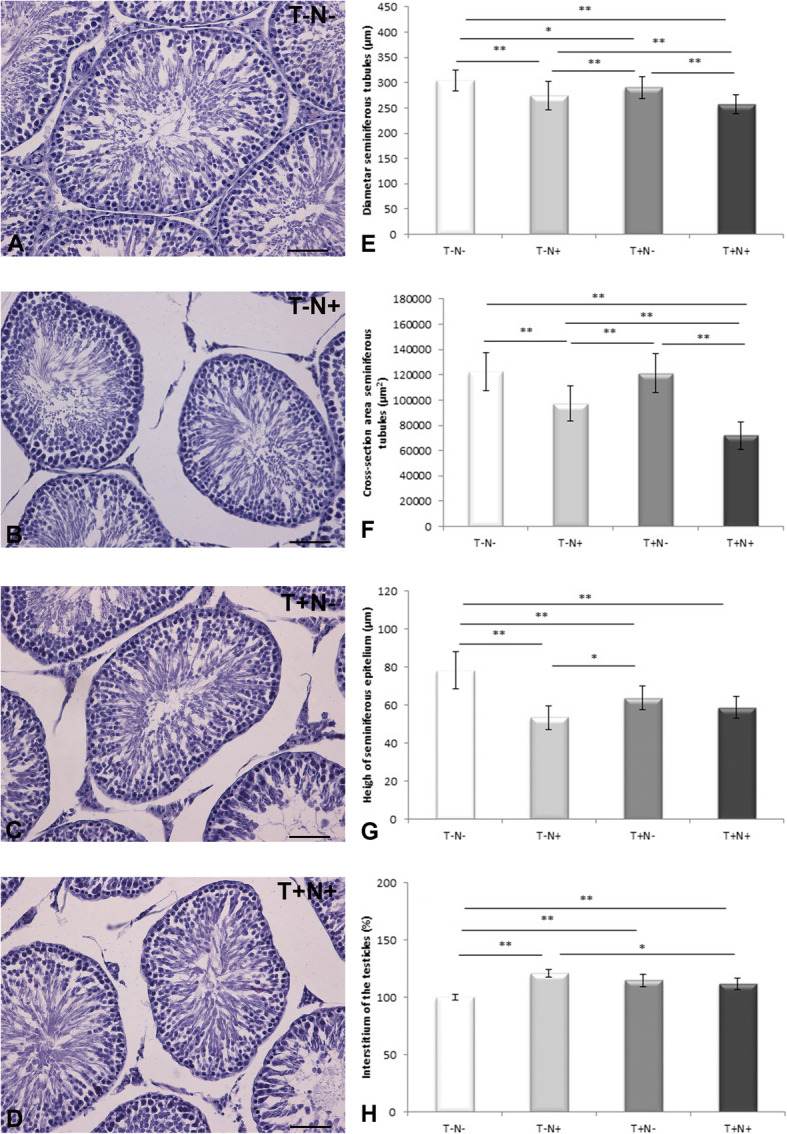
Representative photographs and morphometric parameters of testicular tissue. Left column (**a** to **d**): photographs (magnification 20x; bar = 50 μm) of Hematoxylin and Eosin staining of testicular tissue: **a** control (T-N-), **b** nandrolone group (T-N+), **c** swimming training group (T+N-) and **d** swimming training with nandrolone (T+N+). Right column (**e** to **h**): **e** diameter of the seminiferous tubules, **f** cross-section area of seminiferous tubules, **g** height of seminiferous epithelium, **h** interstitium of the testicle. Results represent as mean values ± SD (*n* = 8). Result for interstitium of the testicle presented in percent. Comparation between groups was performed using one way Anova test with the post-hoc LSD test analysis (* denotes *p*˂0.05; ** denotes *p* < 0.005) (T denotes training, N denotes nandrolone)

Microphotographs of the testicles tissue in the control group showed regular morphological structure without degenerative changes. Nandrolone administration alone (T-N+) or combined with swimming training (T+N+) induced a decrease of the diameter of the seminiferous tubules, the height of seminiferous epithelium as well as expansion of the interstitum area of the testicles. Also, degenerative changes (necrosis, picnosis of the nucleus) were verified in the seminiferous tubules. However, in the swimming training group (T+N-), expansion of the interstitium area of the testicles also occurred. A lower height of seminiferous epithelium was also observed in the swimming training group (T+N-) (Fig. 1a-d).

After nandrolone administration alone, diameter of the seminiferous tubules showed reduction by 10 % (*p* < 0.005), in swimming alone group by 4 % (*p* < 0.05) and in their combination by 15 % (*p* < 0.005) compared to control values. Nandrolone administration alone or combined with swimming training decreased tubule diameter by 6 % (*p* < 0.005) and 12 % (*p* < 0.005) (respectively) compared to swimming training alone. In T+N+ group, diameter was decreased by 6 % (*p* < 0.005) compared to T-N+ group (Fig. 1e).

The most significant reduction of the tubule cross-section area was observed in T-N+ and T+N+ groups (by 21 % (*p* < 0.005) and 41 % (*p* < 0.005) respectively) compared to the control values. Comparison between experimental groups showed that nandrolone alone caused a reduction of the cross-section area of the semeniferous tubules by 25 % (*p* < 0.005) and in combination with swimming by 45 % (*p* < 0.005) in comparison with swimming alone. Combined administration of nandrolone and swimming training decreased cross-section area by 26 % (*p* < 0.005) compared to nandrolone alone (Fig. 1f).

Height of seminiferous epithelium was decreased in T-N+ group by 32 % (*p* < 0.005), in T+N- group by 19 % (*p* < 0.005) and in T+N+ group by 25 % (*p* < 0.005) in comparison to control value. Nandrolone alone decreased the height of the seminiferous epithelium by 20 % (*p* < 0.05) in comparison to training alone (Fig. 1g).

Interstitium of the testicles was increased by 21 % (*p* < 0.005) in T-N+ group, in T+N- group by 15 % (*p* < 0.005), and in T+N+ group by 12 % (*p* < 0.005) compared to control. Training in combination with nandrolone decreased interstitium area by 8 % (*p* < 0.05) in comparison to nandrolone alone (Fig. 1h).

### Androgen receptor

**Fig. 2:**
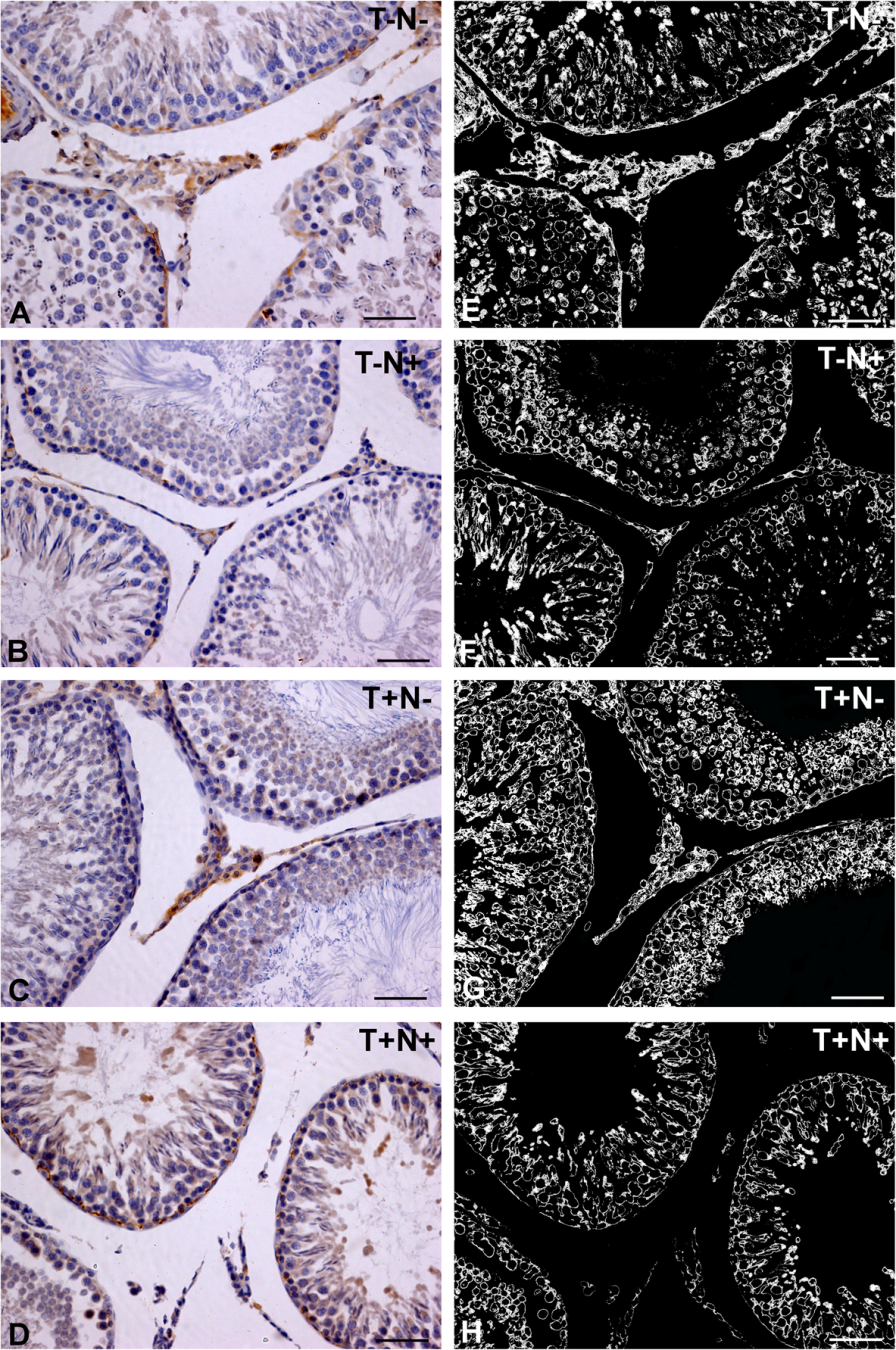
Immunolabelled AR in testicular tissue. Left column (**a** to **d**): photographs (magnification 20x; bar = 50 μm) of immunolabelled AR receptor in testicles: **a**: control (T-N-), **b**: nandrolone group (T-N+), **c**: swimming training group (T+N-) and **d**: swimming training with nandrolone (T+N+) (*n* = 8). Right column (**e** to **h**): Segmented masks (magnification 20x; bar = 50 μm) of AR positive regions in testicles: **e**: control (T-N-), **f**: nandrolone group (T-N+), **g**: swimming training group (T+N-) and **h**: swimming training with nandrolone (T+N+) (AR denotes androgen receptor, T denotes training, N denotes nandrolone)

**Fig. 3:**
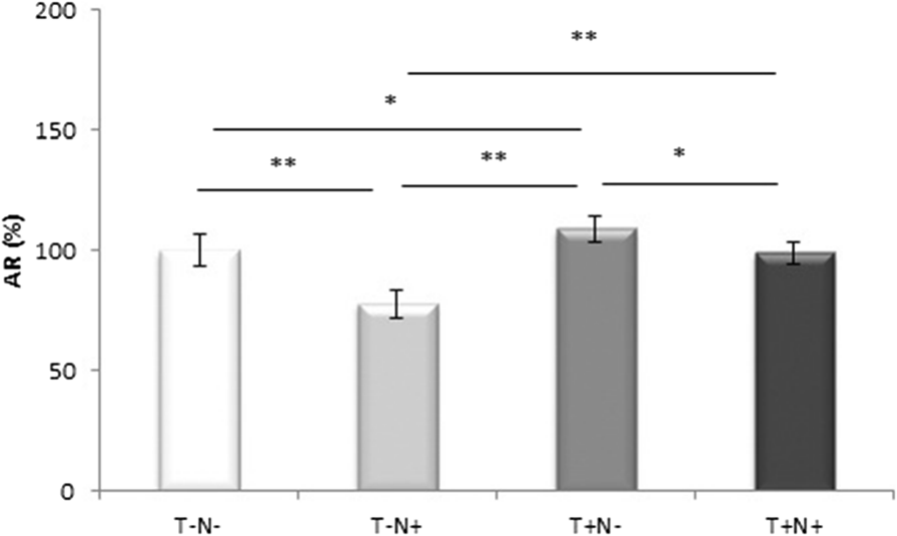
Quantification of AR in testicular tissue. Results represent as mean values ± SD (*n* = 8) presented in percent. Comparation between groups was performed using one way Anova test with the post-hoc LSD test analysis (* denotes *p*˂0.05; ** denotes *p* < 0.005). Control group (T-N-), nandrolone group (T-N+), swimming training group (T+N-) and swimming training with nandrolone group (T+N+), (AR denotes androgen receptor, T denotes training, N denotes nandrolone)

Immunoreactivity of the androgen receptor in the testicles was verified in the Leydig cells, Sertoli cells and myoid peritubular cells of the testicles (Fig. 2a-d) and their localization is shown with a segmented mask of AR positive regions (Fig. 2e-h). In contrast to Leydig and myoid cells, where AR deposits are localized diffusely in the cytoplasm, in Sertoli cells, a completely different pattern of localization was observed. Immunoreactivity to AR has been verified exclusively in the nuclear region. Testicular germ cells were usually negative for AR (Fig. 2a-d). The reduction of the immunoreactivity of androgen receptor was 22 % (*p* < 0.005) in T-N+ group, while there was an increase of 9 % (*p* < 0.05) in T+N- group in comparison to control values. Nandrolone administration alone reduced the immunoreactivity of the androgen receptor by 28 % (*p* < 0.005) and 21 % (*p* < 0.005) in comparison to T+N- and T+N+ groups (respectively). Swimming training alone increased immunoreactivity of the AR by 10 % (*p* < 0.05) compared to T+ N+ group (Fig. 3).

### Redox state

**Fig. 4:**
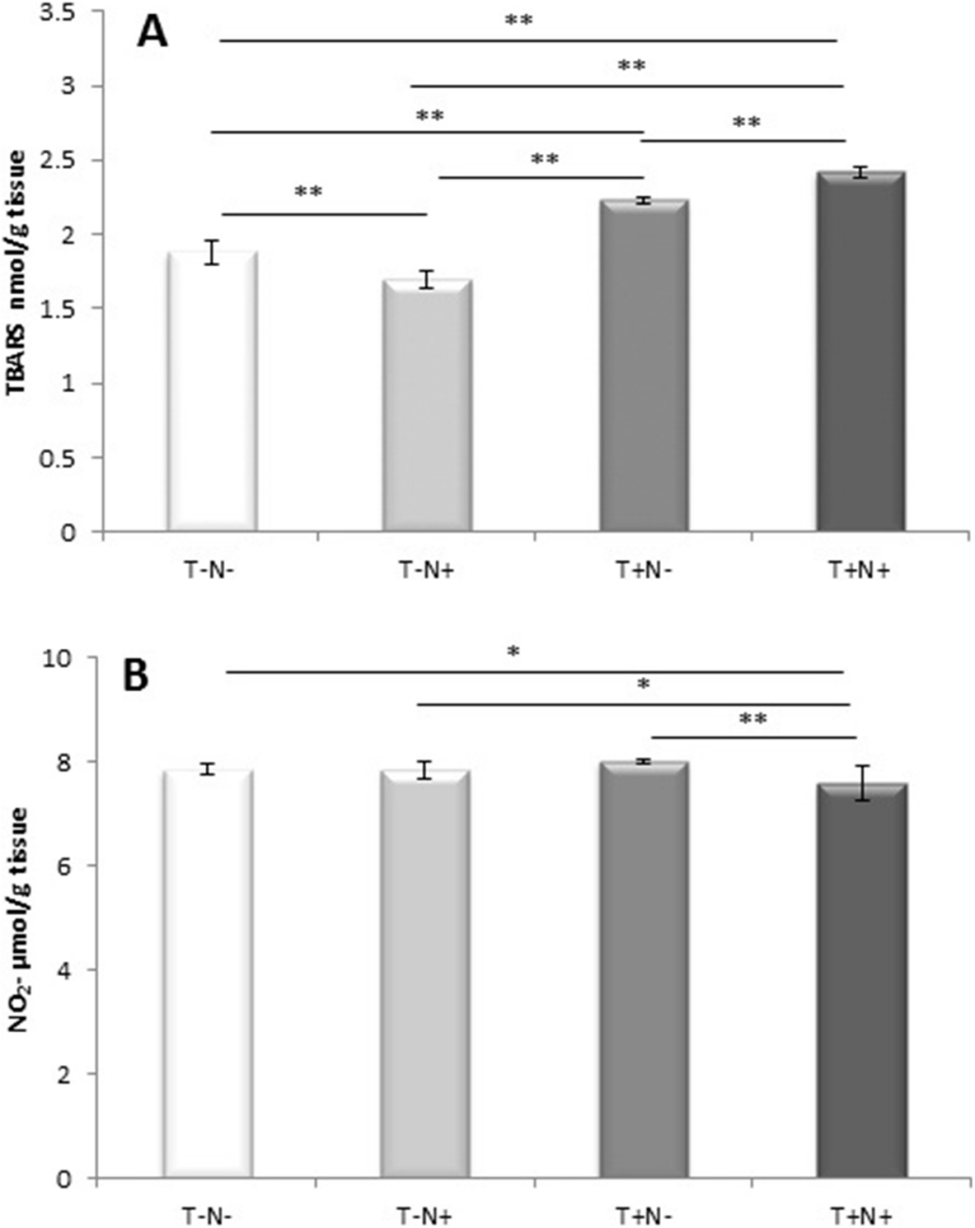
Testicular tissue levels of **a** TBARS and **b** NO_2_^−^. Results presented as mean value ± SD (*n* = 8). Comparation between groups was performed using one way Anova test with the post-hoc LSD test analysis (* denotes *p*˂0.05; ** denotes *p* < 0.005). Control group (T-N-), nandrolone group (T-N+), swimming training group (T+N-) and swimming training with nandrolone group (T+N+), (TBARS denotes index of lipid peroxidation, NO_2_^-^ denotes nitrites, T denotes training, N denotes nandrolone)

**Fig. 5:**
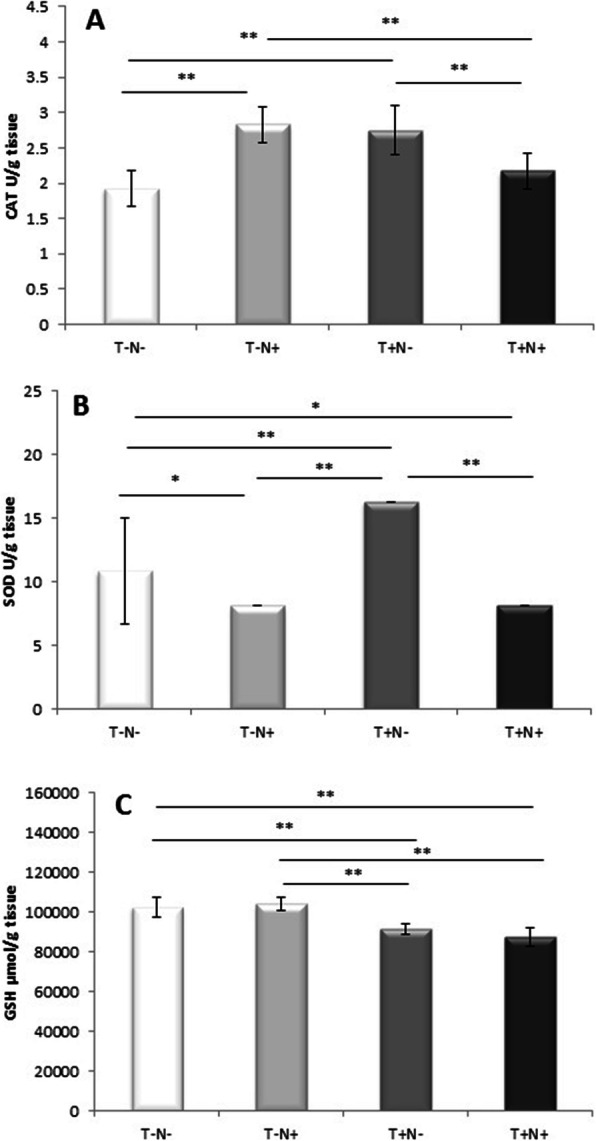
Testicular tissue levels of **a** CAT, **b** SOD, **c** GSH. Results presented as mean values ± SD (*n* = 8). Comparation between groups was performed using one way Anova test with the post-hoc LSD test analysis (* denotes *p*˂0.05; ** denotes *p* < 0.005). Control group (T-N-), nandrolone group (T-N+), swimming training group (T+N-) and swimming training with nandrolone group (T+N+) (CAT denotes catalase, SOD denotes superoxide dismutase, GSH denotes reduced glutathione, T denotes training, N denotes nandrolone)

Index of lipid peroxidation measured as TBARS was reduced after nandrolone administration alone (by 9.5 % (*p* < 0.005)) while swimming training alone or combined with nandrolone increased level of TBARS in the testicles (by 19 % (*p* < 0.005) and by 29 % (*p* < 0.0 5) (respectively)) compared to control (Fig. 4a). Nitrites were significantly altered. Combined administration of nandrolone and swimming training significantly decreased the tissue levels of nitrites compared to control, as well as in the nandrolone alone and swimming training alone groups (Fig. 4b). All experimental protocols increased the level of catalase in the testicles. The largest increase of the catalase levels was observed after nandrolone alone administration (by 47 % (*p* < 0.005)) and swimming alone (by 44 % (*p* < 0.005)) (Fig. 5a) compared to control value. The level of the testicles SOD activity was increased after swimming alone while nandrolone administration alone or combined with swimming decreased SOD in comparison to control (Fig. 5b). The level of the testicle GSH activity was slightly increased after nandrolone administration alone. Oppositely, swimming training alone or combined with nandrolone significantly lowered the GSH acivity in testicles (by 12 % (*p* < 0.005) and by 15 % (*p* < 0.005) (respectively)) compared to nandrolone alone administration (Fig. 5c).

## Discussion

Abuse of AASs, became very popular and widespread among professional and recreative athletes. The main goal of steroid intake is to achieve a rapid muscle mass gain and good looks. However, numerous adverse effects of AASs abuse combined with training on testicular morphology, tissue redox status and regulation of androgen receptor levels are still poorly understood. In the previous study, we have already shown that the nandrolone alone or combined with swimming training decreases volume density and number per mm^2^ of LH and FSH pituitary cells. Now, we wanted to investigate the effects of the mentioned experimental protocol on the testicle tissue in order to complete insight into the pituitary-gonadal axis and the possible role of oxidative stress in the development of reproductive dysfunction.

In this study nandrolone administration alone or combined with swimming reduced testicle weight, e.g. induced testicle atrophy. Similar result was published in other studies in which authors stated that testicular mass is mainly dependent on the mass of spermatogenic cells within the seminiferous tubules [35–38] due to decrease of testosterone level followed by apoptosis of spermatogenic cells and decrease in spermatogenesis [39]. On the other hand, it is known that mild hyperthermia can also reduce testicles weight and provoke apoptosis of germ cells and damage of seminiferous epithelium [40], but in this study, swimming training protocol was designed to minimize morphological changes in testicle tissue caused by temperature [23].

The diameter of the testicular seminiferous tubules is a morphometric parameter which has been used as a good indicator of spermatogenic activity in several studies because direct positive correlation between seminiferous tubules diameter and spermatogenic activity was established [41]. In our study, the diameter of the seminiferous tubules which is in fact related to the height of seminiferous epithelium, was reduced in all experimental groups. These results are consistent with the result of Ferrari et al. who showed that nandrolone alone or in combination with treadmill training reduces the diameter of the testicular seminiferous tubules [41]. They stated that decreased spermatogenic activity and decrease in number of germ cells caused by steroid abuse lead to low-yield spermatogenesis [41]. It can be deduced from this relatively simple parameter and from the findings of serum testosterone levels in the AASs study groups [12] that exogenous steroids induce depletion of the seminal cell population through direct effect on the seminiferous epithelium and through endocrine feedback mechanisms between the pituitary and testicles as well.

An interesting finding is that, even several weeks after the treatment cessation, there is no full restitution of the seminiferous epithelial cell population [42], which reveals the fact that the endocrine mechanisms involved in regulating spermatogenesis are sensitive and susceptible to long-term adverse effects. Similar to the use of AASs, our study showed that intensive training alone leads to a decrease in the diameter of the seminiferous tubules. Since in this case there is no direct adverse effect of AASs, examination of redox status markers in our study showed that oxidative stress interferes with steroidogenesis and spermatogenesis in the testicles [17]. Furthermore, besides the atrophy, microscopic examination of the testicles experimental samples after nandrolone administration alone, degenerative effects such as necrosis, nuclear pycnosis were verified. These very strong morphological effects of AASs can be explained by the findings of studies that showed that nandrolone causes a disruption of the activity of several important enzymes such as 17beta-hydroxylase and 3beta hydroxylase and also DNA damage via antioxidants and apoptosis vectors [43]. Of particular interest is the finding of congestion of the microvasculature in the testicles because it is previously reported that prolonged physical activity can lead to a decrease in blood flow through the testicles [17]. This is important because testicular Leydig cells do not have a significant ability to deposit larger amounts of testosterone. Testosterone is liposoluble, it easily leaves the cell and its concentration in the blood depends largely on the flow through the testicles [17]. As shown in the study by Tahtamouni et al. who stated that nandrolone treatment at both low (3 mg/kg b.w.) and high (10 mg/kg b.w.) doses resulted in atrophy caused by a small number of germ cells in the seminiferous tubules with large focal surfaces and also a decrease in spermatozoa in the seminiferous tubules lumen which indicate a reduction of process of spermatogenesis [35].

Androgen action in the testicles, as well as in other tissues, is mediated through transcriptional activation of the androgen receptor [39]. In our study, immunopositivity to the androgen receptor (AR) in the testicles was expressed in Leydig cells, Sertoli cells as well as myoid peritubular cells. Four weeks administration of nandrolone decanoate alone or combined with swimming training led to a decrease in the amount of androgen receptor. This result is in line with results from the investigation of Tahtamouni et al. [39]. They showed that a low dose of nandrolone decanoate reduced the amount of AR by 32 % while a high dose reduced the expression of AR by 47 %. In their study, only Sertoli cells showed immunopositivity to AR [39]. Loss of AR activity in Sertoli cells leads to disruption of spermatogenesis that would result in incomplete meiosis and collapse of spermatocytes into haploid spermatids. The decrease in the number of Sertoli cells expressing AR may be the cause of delayed maturation, as well as testicular atrophy [39] and a study performed in the cell culture conditions showed that amount of AR in Sertoli cells (as well as iRNA for AR) is dependent on FSH concentration [44]. Our previous results showed that swimming alone increased FSH values in blood stream as well as immunofluorescent signal in the FSH pituitary cells which is in correlation with the mentioned study but combined exposure to the AASs and swimming in this study increased AR amount in the testicles on a much larger scale. Furthermore, combined exposure that induces FSH rise enables Sertoli cells that possess FSH receptors to respond to androgenic stimuli [45], but this is not the case for Leydig and myoid testicular cells. The same study showed that younger animals were more susceptible to the FSH-induced androgenic response compared to adult ones because of the effect of FSH-stimulated AMP phosphodiesterase which in adults diminish the role of FSH [45]. As a proof that very complex regulatory mechanisms are involved in this processes, one study exposed the important role of paracrine factors secreted by Sertoli cells [44]. Indirect proof of FSH-related effect on target tissues AR amount can be found in the study of Willoughby et al. that showed that resistance training increased expression of AR in skeletal muscle and serume testosterone level. High level of testosterone increased the expression of AR in skeletal muscle by up-regulation which in turn causes the increase of myofibrillar proteins in the striated muscle tissue [46].

When reproductive dysfunction is in stake, besides the pituitary-gonadal axis, oxidative stress plays one of the most important roles [17]. Due to the high amount of polyunsaturated fatty acids, testicles are very susceptible to oxidative damage. In our study index of lipid peroxidation in the testicles tissue (measured as TBARS) was increased after swimming training alone or combined with nandrolone decanoate. Similar result was reported by Manna et al. They measured tissue level of malondialdehyde (MDA) and they have shown that swimming increases the level of MDA. These findings can be explained by the inhibition of the steroidogenic enzymes which leads to the increase in lipid peroxidation values in the testicular tissue and consequent inhibition of spermatogenesis induced by the high levels of MDA [23]. Similarly, certain studies have shown a positive correlation between testosterone levels and tissue MDA levels in the left ventricle myocardium. Namely, in cardiomyocytes, testosterone increases the activity of hormone sensitive lipase and thus leads to an increase of lipolysis in cardiomyocytes [47]. In our previous study we already showed that swimming alone or combined with nandrolone led to high levels of testosterone, but in this study we added the finding of increased TBARS levels in testicle tissue which is the proof of a strong correlation between testosterone and TBARS in testicles. We assume that hormone-sensitive lipase is also responsible for increased lipolysis in the testicle tissue, which in turn has led to an increase in tissue values of TBARS. The interesting result of this study is related to the values of nitrites. Having in mind the literature data, we expected that nandrolone would lead to the increase of the nitrites in testicles, but our result is totally opposite. Nandrolone alone or combined with swimming decreased nitrites levels in testicle tissues. Ahmed et al. have shown that nandrolone increased the level of NO in testicles in a similar study design. They stated that the increase in NO in the testicles can be explained by the increase in nitric oxide synthase formed as a consequence of nandrolone administration [35]. Nitric oxide led to inhibition of steroidogenesis as well as testosterone secretion [35, 48]. The reason for this disagreement is not easy to find unless some methodology approach caused such difference.

Another oxidative stress marker wich is the important part of cell protection from lipid peroxidation, as well as from ROS production is SOD. In our study nandrolone administration alone decreased levels of SOD in the testicle tissues which is in line with literature data [9, 35]. Inhibition of tissue SOD activity may result in the accumulation of superoxide anion radicals and later lipid peroxidation [3, 49]. Swimming training alone increased levels of SOD activity which is opposite from results from Manna [17]. CAT activity was increased after nandrolone alone administration and swimming training alone or combined with nandrolone. This result is opposite from literature data [17]. By joint action of SOD and CAT activity, superoxide anion radical (O_2_) will be removed. This process has an important part in the reduction of oxidative stress and lipid peroxidation [23].

Glutathione represents an endogenous antioxidant, and has an important role in the process of removing free radicals and thus regulating the redox status of the cells [17, 50]. It is known that Sertoli cells could provide GSH as a source of cysteine which gives a better ability of spermatogenic cells to reduce free radicals [51]. In the present study, tissue GSH activity levels were slightly increased after the administration of nandrolone, which is consistent with the results from the study of Tahtamouni et al. [39]. They have shown that both low and high doses of nandrolone slightly increase GSH levels in testicular tissue and other antioxidant mechanisms such as SOD, catalase, GPx to eliminate oxidative stress are used [39]. Opposite to this, Ahmed et al. showed that nandrolone administration during 8 weeks, decreased the level of GSH in testicles. The decreased levels of tissue GSH can be considered as a marker of oxidative stress [35], and can also lead to decreased spermatogenesis because Sertoli cells metabolized GSH to amino acids used in the process of spermatogenesis [36, 51]. In our study swimming training alone decreased the level of GSH which is in line with results from Manna [17]. The decrease in the value of GSH indicates increased production of free radicals [50], which coincides with our result. This result suggests that swimming alone or in combination with nandrolone may lead to disruption of spermatogenesis.

## Conclusions

**Fig. 6:**
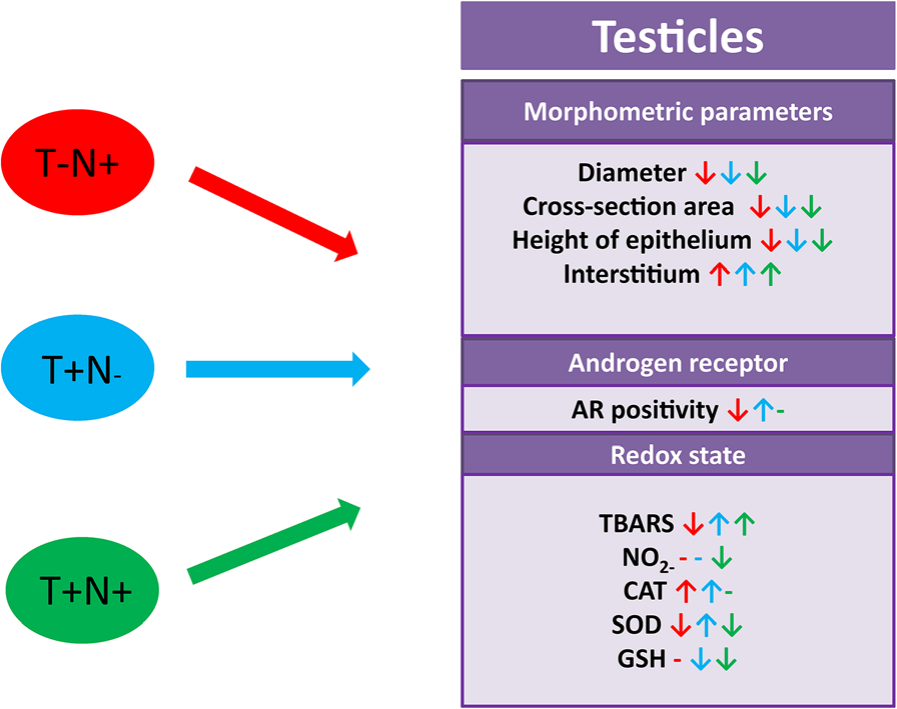
Influence of applied protocols on different parameters observed in this study. Morphometric parameters showed in this figure: diameter, cross-section area and height of epithelium refer to seminiferous tubules. Nandrolone group (T-N+), swimming training group (T+N-) and swimming training with nandrolone group (T+N+). **↓** represents decrease; ↑ represents increase; - represents no alteration in comparison to control group (T-N-) (T denotes training, N denotes nandrolone)

We concluded that supraphysiological doses of nandrolone, applied alone or in combination with swimming training impaired histomorphometric parameters and decreased the amount of AR in the rat testicles. Combined administration of nandrolone and swimming training caused the most prominent changes in redox homeostasis in testicle tissue, which is an indicator/cause of impaired reproductive function (Fig. 6). These results reinforce the severity of AASs abuse effects in combination with physical exercise considering reproductive health. In addition, more research is needed to further elucidate complex mechanisms of these negative effects.

## Data Availability

The datasets used and/or analysed during the current study are available from the corresponding author on reasonable request.

## Abbreviations

AASs: Anabolic androgenic steroids
AR: Androgen receptor
AMP: Adenosine monophosphate
CAT: Catalase
DAB: Diaminobenzidine
DNA: Deoxyribonucleic acid
DPX: Dibutylphthalate Polystyrene Xylene
EDTA: Ethylenediaminetetraacetic acid
FSH: Follicle stimulating hormone
GSH: Reduced glutathione
HRP: Horseradish peroxidase
LH: Luteinizing hormone
MDA: Malonaldehyde
N: Nandrolone
NO_2_^−^: Nitrites
NO: Nitric oxide
PBS: Phosphate buffer saline
PCA: Perchloride acid
ROS: Reactive oxigen species
SOD: Superoxide dismutase
T: Training
TBA: Thiobarbituric acid
TBARS: Index of lipid peroxidation
PBS: Phosphate buffer
